# First-Order Statistic of Afterpulsing and Crosstalk Events in Silicon Photomultipliers

**DOI:** 10.3390/s26144432

**Published:** 2026-07-13

**Authors:** Sergey Vinogradov

**Affiliations:** P.N. Lebedev Physical Institute of the Russian Academy of Sciences, Leninskiy Prospekt 53, Moscow 119991, Russia; vinogradovsl@lebedev.ru

**Keywords:** silicon photomultiplier, SiPM, afterpulsing, crosstalk, Poisson process, arrival time, order statistic

## Abstract

This paper presents an order statistic approach to the time distribution of the first detected event following a primary avalanche pulse, considering a mixture of correlated and primary dark counts. The well-known order statistic method, commonly used to describe the time resolution of scintillation detectors, is applied to the arrival times of correlated events. The established model of crosstalk as a branching Poisson process is extended to afterpulsing, and correlated events are analyzed starting from their initial seeds—free (detrapped or diffused) charge carriers capable of triggering secondary avalanche breakdowns. The proposed approach enables the extraction of true timing information for delayed crosstalk and afterpulsing and predicts a narrowing of the first arrival time distribution as the number of seeds and the probability of correlated events increase.

## 1. Introduction

Afterpulsing (AP) was first identified in photomultiplier tubes (PMTs) in the mid-1950s as a source of correlated noise caused mainly by positive-ion feedback [1,2]. This noise appears as stochastic false signals in the form of delayed single electron response pulses produced by an initial primary signal. AP affects PMT performance especially in low-light and photon counting applications, for example, in astrophysics, resulting in excess background and false signal counts [3,4].

AP is commonly characterized by the probability of the occurrence of at least one AP pulse (referred to as an event) and by the delay time after the primary event. The probability distribution of AP arrival times in PMT reflects several complex processes, including ion feedback resulting from the ionization of residual gas atoms and molecules within the PMT volume and at the dynode surfaces, cathodoluminescence from the dynode surfaces, transition-radiation emission, and other phenomena [5]. The probability density function (PDF) of arrival times f(t) has been modeled using multiexponential decay and Laguerre-type polynomial functions [6]. In the most typical cases, AP events are produced by different types of ions under an ion-feedback mechanism, generating multipeak arrival-time histograms that are modeled using a multi-Gaussian PDF [7].

The AP effect was also observed in another type of high-gain photodetector, the Geiger-mode avalanche photodiode (GM APD), a decade later than in PMTs. The GM APD operates at a bias voltage above the breakdown voltage; therefore, a single charge carrier entering an avalanche region can trigger a breakdown process of, in principle, unlimited gain. In practice, the gain is limited by a quenching process governed by the passive or active circuit of the GM APD. GM APDs are more efficient and accurate in detecting single photons compared to PMTs, so they are commonly called single photon avalanche diodes (SPADs). AP in GM APDs or SPADs was attributed to the re-emission, or detrapping, of charge carriers captured at deep-level sites in the semiconductor during a primary avalanche breakdown [8,9].

SPAD functionality depends heavily on AP, because true photon detection and false AP detection are identical events, which can be differentiated only by their statistics. AP plays a critical role in the degradation of SPAD performance in optical quantum communications (increasing bit error rates and limiting data rates), time-of-flight laser ranging, optical time-domain reflectometry, time-correlated single-photon counting, and other application areas [10,11]. On the other hand, AP measurements enable comprehensive studies of semiconductor defects at the level of single electron trapping–detrapping processes, as considered in [9,12].

The silicon photomultiplier (SiPM) is an analog device based on the concept of noiseless amplification of a single charge carrier, combining the Geiger-mode avalanche breakdown as a positive feedback process and passive quenching as a negative feedback process dependent on the avalanche charge [13]. The higher the excess bias above the breakdown voltage, the stronger the positive feedback and the higher the gain, and, simultaneously, the stronger the negative feedback, the faster the quenching, and the lower the excess noise factor (ENF) of the avalanche multiplication. A common SiPM design is an array of SPAD pixels with passive quenching by in-pixel resistors. Pixels are operated independently of each other and connected in parallel to a common cathode and anode. Therefore, an output signal from the SiPM is the sum of all signals from all pixels [14,15,16]. The SiPM provides proportional detection of low-level light pulses starting from single photons with excellent photon number resolution and good time resolution at room temperature. Since the mid-2000s, SiPMs have been recognized worldwide as new photon detectors that are highly competitive with conventional PMTs and APDs.

The SiPM inherits all AP-related features of a single SPAD. Moreover, the SiPM, as an array of SPAD pixels, exhibits another type of correlated noise, crosstalk (CT). CT events occur if a primary fired pixel (primary event) ignites other pixels of the array. The CT effect in SPAD arrays and SiPMs is caused by the so-called hot carrier luminescence, namely, emission of photons during avalanche breakdown due to various mechanisms of radiative losses of charge carrier energy, such as bremsstrahlung [17,18]. Thus, neighboring pixels can detect these secondary photons, and this effect is often referred to as optical CT. There are two types of optical CT distinguished by their delay time. The prompt CT pulse occurs instantly, overlapping with the primary one, so its delay time is hardly resolvable because of the picosecond-scale drift of photoelectrons or holes produced by absorption of secondary photons in a depleted region of the pixel. The delayed CT is typically associated with nanosecond-scale diffusion of the photoelectrons or holes from an undepleted region of the pixel.

CT and AP represent an essential drawback in SiPM performance because the photon number resolution and arrival time resolution of incident light pulses are degraded by the specific ENF of correlated events. This problem appears in many SiPM application areas such as high energy physics (particle calorimeters of CERN LHC, FermiLab), astrophysics (gamma-imaging Cherenkov telescopes), nuclear medicine (TOF PET, SPECT detectors), and others. To account for the contribution of correlated noises and optimize the detection, we need to know, in general, the probability distribution of the number of correlated events over the delay time. The first theoretical models of geometric and Borel distributions have been developed and verified for the simplest case of prompt CT events [19]. However, delayed CT and AP are time-dependent processes and thus more complicated. There have been just a few attempts to describe an infinite sequence of afterpulses created by afterpulses, and they did not result in convenient expressions (see, for example, [20]). Therefore, most theoretical and experimental studies consider the probability distribution of arrival time of the first correlated event. Nevertheless, this simplified approach provides useful information about the probability of occurrence and characteristic time of delayed CT and AP events, which might be sufficient to understand their origins, develop correction measures, and optimize detection performance.

A widely used model of the probability density function of the AP arrival times in SPADs is the multiexponential PDF $f_{exp}\left(t\right)$ [9]:

(1) $$f\left(t\right)=f_{exp}\left(t\right)=\sum_{i}p_{i}\cdot \frac{\exp(-t/\tau_{i})}{\tau_{i}}$$

where the index *i* = 1, 2… denotes the contributing arrival processes with probabilities $p_{i}$ and detrapping time constants $\tau_{i}$, presumably associated with monoenergetic trap levels.

Most studies of AP in SiPMs have employed this model (1) to extract either a single time constant [21] or two (fast and slow) time constants [22,23].

Initial studies of AP in Geiger-mode APDs based on III-V compound heterostructures, such as InGaAs/InP, also used a single-exponential model (1) [24]. Subsequent studies, however, showed that the experimental distributions could be described by a power-law function $f_{pow}\left(t\right)∼t^{-\alpha }$. A fixed value of α provided a substantially better fit to the experimental data than a multiexponential function with three or more components [25]. The authors assumed that this power-law decay arises from a broad spectrum of trap energy levels in InP, which produces a distribution of detrapping time constants. They further suggested that these constants could be broadened by the Poole–Frenkel effect in the strong, nonuniform electric field within the avalanche region.

The dependence of AP PDF on the deep-level energy spectrum was further analyzed by Horoshko et al. [26]. Assuming a quasi-continuous distribution of deep levels with constant density over a defined energy range, they derived a hyperbolic-sinc model, $f_{sinc}\left(t\right)$. They showed that the power-law function $f_{pow}\left(t\right)$ is a close approximation of $f_{sinc}\left(t\right)$ and that both models agree well with the experiments.

The comparative evaluation study of the $f_{exp}$, $f_{pow}$, and $f_{sinc}$ models using experimental data from several widely used SPAD, including Excelitas SPCM and Laser Components τ-SPAD, resulted in the following conclusion [27]: *“Our data clearly proves that none of the current theoretical models are universal which makes it hard to draw conclusions about the underlying mechanism based on fundamental semiconductor physics.”*.

In addition to detrapping, the diffusion of minority carriers has been identified as another source of delayed correlated events. Secondary photons emitted during a primary avalanche can generate minority carriers in undepleted bulk silicon. Their subsequent diffusion can produce delayed crosstalk (CT) in neighboring pixels or retrigger the initially fired pixel, resulting in the so-called optically-induced AP ([28,29], see also ICASiPM-2018 [30]). This second type of AP can be considered in terms of timing in the same way as the first type of AP related to detrapping; therefore, we do not need to differentiate them in contrast to prompt and delayed CT. In this case, a single time constant corresponding to the minority-carrier lifetime in the silicon substrate provides a good fit to the experimental data for both delayed CT and AP [31].

Mostly focused on the specific generation and transport mechanisms responsible for the AP time profile, many existing AP timing models share a common assumption: a primary avalanche generates a single secondary avalanche with a certain probability. Under this assumption, the occurrence of AP is governed by a Bernoulli process, whereas the AP time profile is determined by the transit time or arrival-time distribution of the charge carrier that triggers the secondary avalanche. This description corresponds to a geometric chain process in which each event produces either no successor or a single successor [19].

An alternative scenario arises when a primary avalanche generates multiple free charge carriers in a depleted region due to detrapping or diffusion and, consequently, a random number of seeds. These seeds compete to be the first to reach an avalanche region and trigger a pixel. The timing of the first detected event must, therefore, be described using order statistics. Such approaches are commonly used to model scintillation detector time resolution [32,33,34].

The similarity between delayed CT and AP, particularly when both arise from carrier diffusion, motivates the use of a branching Poisson-process framework [19]. This framework is widely used to describe prompt CT distributions [35,36,37]. For a branching Poisson process initiated by a single primary avalanche, the number of direct descendants in a single branch follows a Poisson distribution, whereas the total number of events follows a Borel distribution. We extend this framework by assuming that the primary avalanche generates a Poisson-distributed number of first-generation seeds having a common arrival-time (transit time) distribution.

The resulting multi-seed model differs considerably from the single-event geometric chain models when the probability of correlated events is high. This difference is particularly relevant in at least three cases: (i) early-stage SiPM and SPAD development, when delayed CT and AP have not yet been adequately suppressed and their probabilities could be as high as ≈45% (first series of Hamamatsu MPPC S10362 devices [23]) and ≥80% (SPAD development at Toshiba Corp. [38]); (ii) low-temperature SiPM applications, in which self-sustaining AP “trains” have been observed [39,40]; and (iii) radiation-hard SiPM applications, in which irradiation-induced silicon defects can cause, for example, an eightfold increase in trapping-induced AP even at a neutron fluence as low as $3.3\cdot 10^{9}$ neq/cm^2^ [41]. At a higher fluence of approximately $10^{12}$ neq/cm^2^, AP becomes masked by dark counts [42].

This paper presents a first-order statistic approach to the time distribution of the first detected event initiated by a random number of Poisson-distributed seeds in the first generation following a single primary event. The order statistic defines the dependence of the arrival time distribution on the number of seeds, contrasting with the geometric chain process, where the appearance of AP and its timing profile are independent. The multi-seed framework also provides a unified description of prompt and delayed CT timing. Finally, we discuss earlier papers that incorporated some assumptions on the Poisson distribution of delayed correlated events and their arrival time models [22,31,42,43].

## 2. First-Order Statistic of Correlated and Primary Dark Counts

The method is based on the following assumptions:

1. A primary avalanche event generates a random number of seeds *N* following a Poisson distribution with mean μ=E[N].
2. The seed arrival times are independent and identically distributed random variables $T_{n}$, *n* = 1, 2 … *N*, with cumulative distribution function (CDF) $F\left(t\right)=Pr\left(T_{n}\leq t\right)$ and probability density function (PDF) f(t)=dF(t)/dt.
3. Each seed triggers a secondary avalanche, referred to as a correlated event, through a Bernoulli process with probability $P_{trig}$.
4. Multiple types of correlated processes, characterized by distinct arrival-time distributions, mean seed numbers, and triggering probabilities, may coexist independently.
5. Correlated processes mixed with the primary dark counting process are considered independent Poisson point processes.
6. A Poisson point process produces only event timestamps $T_{n}$ (i.e., pulse amplitudes and shapes are not attributed to the event). The process is fully characterized by its mean number of events N(t) or equivalently by its intensity I(t)=dN(t)/dt.
7. Primary dark counting is a homogeneous (stationary) process with constant intensity DCR.
8. Correlated processes are inhomogeneous (nonstationary) with time-dependent intensities.

Our objective is to derive the probability distribution of the arrival time $T_{min}$ of the first event generated by superposition of these processes.

Consider two independent random variables $T_{C}$ and $T_{D}$, representing the arrival times of the first correlated and primary dark events, respectively. The random variable $T_{min}=\min\left[T_{C},T_{D}\right]$ has CDF $F_{min}\left(t\right)$. The probability theory of order statistics provides expressions for the CDF and PDF for any order of ordered (sorted) random variables. In first-order statistics, the distribution of the minimum is most conveniently derived using the complementary CDF (CCDF) (survival function). For a random variable *T*, the events (T≤t) and (T>t) are mutually exclusive and the respective probabilities are complementary (Pr(T≤t)+Pr(T>t)=1); therefore, Pr(T>t)=1−F(t). Because the absence of a correlated event and the absence of a primary dark event during the interval (0,t) are independent, the probability of no event from both processes is the product of $CCDF_{C}$ and $CCDF_{D}$:

(2) $$\begin{array}{cc} CCDF_{min} & =CCDF_{C}\cdot CCDF_{D} \end{array}$$

(3) $$\begin{array}{cc} 1-F_{min}\left(t\right) & =\left(1-F_{C}\left(t\right)\right)\cdot \left(1-F_{D}\left(t\right)\right) \end{array}$$

(4) $$\begin{array}{cc} F_{min}\left(t\right) & =1-\left(1-F_{C}\left(t\right)\right)\cdot \left(1-F_{D}\left(t\right)\right) \end{array}$$

If $T_{D}$ is the arrival time of the first dark event from a Poisson point process with intensity DCR, then

(5) $$\begin{array}{cc} F_{D}\left(t\right) & =1-\exp(-DCR\cdot t) \end{array}$$

(6) $$\begin{array}{cc} F_{min}\left(t\right) & =1-(1-F_{C}\left(t\right))\cdot \exp\left(-DCR\cdot t\right) \end{array}$$

(7) $$\begin{array}{cc} f_{min}\left(t\right) & =(f_{C}\left(t\right)+DCR\cdot \left(1-F_{C}\left(t\right)\right))\cdot \exp\left(-DCR\cdot t\right) \end{array}$$

Equation (6) makes it possible to extract the probability distribution $F_{C}\left(t\right)$ of correlated events of our interest from measurements of mixed processes, as demonstrated in [44].

Next, we consider a correlated process *C*, in which a primary avalanche generates a random number *N* of seeds capable of producing a secondary avalanche. To find the marginal (unconditional) distribution of the first seed arrival time $T_{min}=\min\left[T_{1},T_{2}\ldots T_{N}\right]$, we sum the conditional CDFs $Pr(T_{n}\leq t|N=n)$ for a fixed *n* over all possible values of *N*, weighted by Poisson probability Pr(N=n):

(8) $$\begin{array}{cc} F_{min}\left(t\right) & =\sum_{n=1}^{\infty }Pr\left(T_{n}\leq t|N=n\right)\cdot Pr\left(N=n\right)\\ \; & =\sum_{n=1}^{\infty }\left[1-{\left(1-F_{C}\left(t\right)\right)}^{n}\right]\cdot \frac{\mu^{n}\cdot e^{-\mu }}{n!} \end{array}$$

The sum in Equation (8) has the closed form [45]:

(9) $$F_{min}\left(t\right)=1-\exp\left(-\mu \cdot F_{C}\left(t\right)\right)$$

The PDF of the distribution in Equation (9) is

(10) $$f_{min}\left(t\right)=\mu \cdot f_{C}\left(t\right)\cdot \exp\left(-\mu \cdot F_{C}\left(t\right)\right)$$

where $\mu \cdot f_{C}\left(t\right)$ is the intensity of the inhomogeneous Poisson process of seed arrivals.

A correlated event occurs when an arriving seed triggers a secondary avalanche with probability $P_{trig}$. This Bernoulli process results in the random splitting of seed arrivals into triggered events with a mean $\mu_{trig}=P_{trig}\cdot \mu$ and the absence of events with a mean $\mu_{no-trig}=\left(1-P_{trig}\right)\cdot \mu$. This splitting or thinning of the Poisson process modifies the intensity of the Poisson arrival process $\mu \cdot f_{C}\left(t\right)$ to $I_{C}=P_{trig}\cdot \mu \cdot f_{C}\left(t\right)$. In general, different correlated processes may have different triggering probabilities $P_{trig}$ because their sources differ in spatial location relative to the avalanche p-n junction [46,47].

Prompt crosstalk need not be excluded from the model, although its timing is generally unresolvable from that of the primary event. In this case, its arrival-time PDF $f_{pCT}\left(t\right)$ can be approximated by a Dirac delta function. Therefore, the total intensity of the crosstalk processes $I_{CT}\left(t\right)$ is the sum of the prompt $I_{pCT}\left(t\right)$ and delayed $I_{dCT}\left(t\right)$ components with corresponding mean numbers of seeds, transit time functions, and constant triggering probabilities:

(11) $$\begin{array}{cc} I_{CT}\left(t\right) & =I_{pCT}\left(t\right)+I_{dCT}\left(t\right)\\ \; & =P_{trig-pCT}\cdot \mu_{pCT}\cdot f_{pCT}\left(t\right)+P_{trig-dCT}\cdot \mu_{dCT}\cdot f_{dCT}\left(t\right) \end{array}$$

For AP events, pixel recovery must be incorporated through a time-dependent triggering probability $P_{trig-AP}\left(t\right)$:

(12) $$I_{AP}\left(t\right)=P_{trig-AP}\left(t\right)\cdot \mu_{AP}\cdot f_{AP}\left(t\right)$$

Here, $P_{trig-AP}\left(t\right)=P_{trig-AP}\left(V_{ov}\left(t\right)\right)$, where $V_{ov}\left(t\right)$ is the actual overvoltage, which appears to be a time-dependent function during the recovery of pixels. The functions $P_{trig}\left(V_{ov}\right)$ and $V_{ov}\left(t\right)$ can be determined from experimental measurements or from models of triggering probabilities and recovery dynamics, as discussed elsewhere [29,46].

Finally, the superposition of independent Poisson processes is itself a Poisson process, for which we obtain expressions for the CDF $F_{min}$ and PDF $f_{min}$ of the first observed event, defined solely by the total process intensity $I_{sum}\left(t\right)$:

(13) $$\begin{array}{cc} & I_{sum}\left(t\right)=I_{CT}\left(t\right)+I_{AP}\left(t\right)+DCR \end{array}$$

(14) $$\begin{array}{cc} & N_{sum}\left(t\right)=\int_{0}^{t}I_{sum}\left(t^{\prime }\right)dt^{\prime } \end{array}$$

(15) $$\begin{array}{cc} & F_{min}\left(t\right)=1-\exp\left(-N_{sum}\left(t\right)\right) \end{array}$$

(16) $$\begin{array}{cc} & f_{min}\left(t\right)=I_{sum}\left(t\right)\cdot \exp\left(-N_{sum}\left(t\right)\right) \end{array}$$

Note that all expressions in this paper are derived under the assumption of temporal point processes. When necessary, they should be modified to account for specific event amplitudes, pulse shapes, and discrimination conditions. Under such conditions, some correlated pulses may be lost during the initial time interval if they are not resolved from the primary pulse or rejected due to reduced amplitude during pixel recovery.

## 3. Discussion

### 3.1. Comparison of Branching Poisson and Geometric Chain Models

We first compare first-arrival time distributions in the absence of dark counts for two models: a geometric chain process, which produces a single correlated event with probability *p*, and the first generation of a branching Poisson process, which produces a Poisson-distributed number of correlated events with mean $\mu_{trig}$. For clarity, we consider the simplest case: a single-exponential arrival-time distribution $T_{exp}$ with PDF $f_{exp}\left(t\right)$, a single type of correlated process, such as delayed crosstalk with $P_{trig}\left(t\right)=const$, and a dark count rate DCR=0.

In the geometric chain model, the arrival time of the first correlated event $T_{min-geom}$ coincides with the arrival time $T_{geom}$ of its single possible event. The corresponding CDF and PDF of $T_{geom}$ are therefore given by the product of the Bernoulli probability *p* and the CDF and PDF of the exponential arrival-time (transit time) distributions $F_{exp}\left(t\right)$ and $f_{exp}\left(t\right)$.

In the Poisson model, the CDF and PDF of the first event arrival time $T_{min-pois}$ are obtained from Equations (9) and (10) with the mean number of triggered events $\mu_{trig}$.

(17) $$\begin{array}{cc} f_{exp}\left(t\right) & =\frac{e^{-t/\tau }}{\tau } \end{array}$$

(18) $$\begin{array}{cc} F_{exp}\left(t\right) & =1-e^{-t/\tau } \end{array}$$

(19) $$\begin{array}{cc} f_{min-geom}\left(t\right) & =f_{geom}\left(t\right)=p\cdot f_{exp}\left(t\right)=p\cdot \frac{e^{-t/\tau }}{\tau } \end{array}$$

(20) $$\begin{array}{cc} F_{min-geom}\left(t\right) & =F_{geom}\left(t\right)=p\cdot F_{exp}\left(t\right)=p\cdot \left(1-e^{-t/\tau }\right) \end{array}$$

(21) $$\begin{array}{cc} f_{min-pois}\left(t\right) & =\mu_{trig}\cdot f_{exp}\left(t\right)\cdot e^{-\mu_{trig}\cdot F_{exp}\left(t\right)}=\mu_{trig}\cdot \frac{e^{-t/\tau }}{\tau }\cdot e^{-\mu_{trig}\cdot \left(1-e^{-t/\tau }\right)} \end{array}$$

(22) $$\begin{array}{cc} F_{min-pois}\left(t\right) & =1-e^{-\mu_{trig}\cdot F_{exp}\left(t\right)}=1-e^{-\mu_{trig}\cdot \left(1-e^{-t/\tau }\right)} \end{array}$$

Note that both $T_{min-pois}$ and $T_{geom}$ are improper random variables. Their probability functions are therefore termed defective CDF and PDF, because a fraction (1−p) of trials (primary events) in the geometric chain process and a fraction $\exp(-\mu_{trig})$ of trials in the Poisson branching process do not produce correlated events; therefore, their arrival times are undefined. Consequently, their CDFs approach values less than unity at infinite time (Equations (20) and (22)):

(23) $$\begin{array}{cc} F_{geom}\left(t\to \infty \right) & =p \end{array}$$

(24) $$\begin{array}{cc} F_{min-pois}\left(t\to \infty \right) & =1-\exp(-\mu_{trig})=p \end{array}$$

Thus, in the Poisson model, the probability of correlated events *p* is defined by Equation (24) as the probability to get at least one triggered event, which establishes the equivalence of the geometric and Poisson process descriptions [19].

For a low mean number of triggered events ($\mu_{trig}\ll 1$), the two models become nearly identical:

(25) $$\begin{array}{cc} \; & p=1-\exp\left(-\mu_{trig}\right)\approx \mu_{trig} \end{array}$$

(26) $$\begin{array}{cc} \; & f_{min-pois}\left(t\right)\approx \mu_{trig}\cdot f_{exp}\left(t\right)\approx f_{geom}\left(t\right) \end{array}$$

In this case, the most probable non-zero number of arriving events from a Poisson branch is one, matching the single-event structure of the geometric chain process, and the model results are similar.

In contrast, when $\mu_{trig}$ is large, the last exponential factor in Equation (21) significantly compresses the first-arrival time distribution towards shorter times and narrower widths relative to $f_{exp}\left(t\right)$. This effect is well known in multiphoton pulse arrival time measurements, where it appears as a dependence of time walk and timing resolution on the number of photons per pulse.

**Figure 1:**
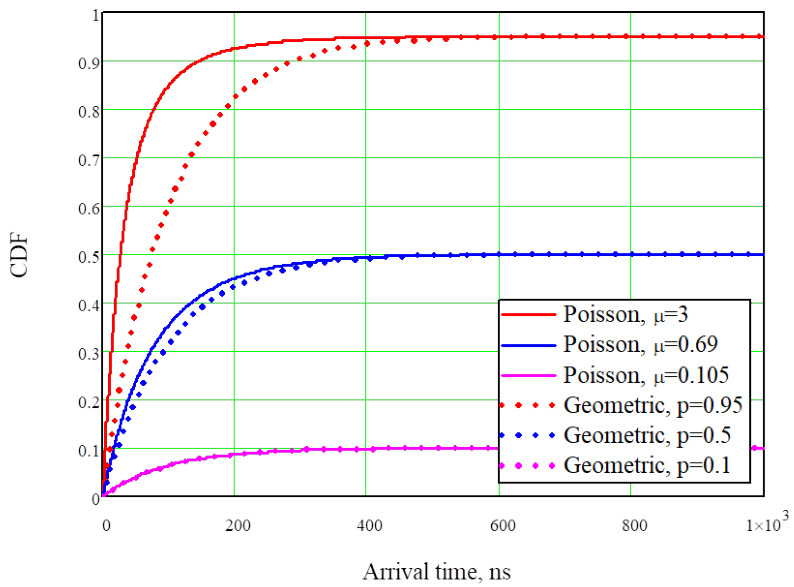
Cumulative distribution functions (CDFs) of the arrival times of the first correlated event from the Poisson branching process, Equation (22), and the single correlated event from the geometric chain process, Equation (20), in the absence of dark counts. The Poisson and geometric processes have the same single-exponential CDF $F_{exp}\left(t\right)$ with a time constant τ = 100 ns, and they have equal correlated event probabilities according to Equations (23) and (24).

**Figure 2:**
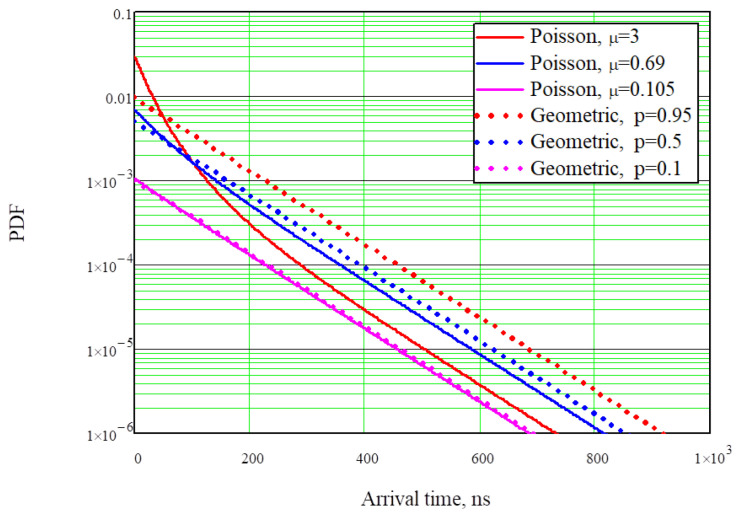
Probability density functions (PDFs) of the arrival times of the first correlated event from the Poisson branching process, Equation (21), and the single correlated event from the geometric chain process, Equation (19), in the absence of dark counts. The Poisson and geometric processes have the same single-exponential PDF $f_{exp}\left(t\right)$ with a time constant τ = 100 ns, and they have equal correlated event probabilities according to Equations (23) and (24).

The comparative behavior of the geometric chain model and the branching Poisson model (Equations (17)–(22)) is shown in Figure 1 and Figure 2 for the same single-exponential PDF $f_{exp}\left(t\right)$ with time constant τ = 100 ns, and the same probabilities of correlated events according to Equations (23) and (24).

As expected, the Poisson and geometric distributions are nearly identical at probability p=0.1, where both PDFs reproduce the single-exponential decay $p\cdot f_{exp}\left(t\right)$ (Equations (19) and (26)). The higher the probabilities, the greater the deviation of Poisson CDFs and PDFs towards earlier arrivals relative to the geometric distributions, which remain simple p− scaled versions of $F_{exp}\left(t\right)$ and $f_{exp}\left(t\right)$, respectively.

We finally consider the first-arrival time distributions when the correlated events and dark counts are mixed. Using the general expression Equation (6) for CDF $F_{min}\left(t\right)$, and substituting an arbitrary correlated process CDF $F_{C}\left(t\right)$ by the specific CDFs $F_{min-geom}\left(t\right)$ (Equation (20)) and $F_{min-pois}\left(t\right)$ (Equation (22)), we obtain the following:

(27) $$\begin{array}{cc} F_{min-geom-dcr}\left(t\right) & =1-\left(1-p\cdot \left(1-e^{-t/\tau }\right)\right)\cdot e^{-DCR\cdot t} \end{array}$$

(28) $$\begin{array}{cc} f_{min-geom-dcr}\left(t\right) & =\left(p\cdot \frac{e^{-t/\tau }}{\tau }+DCR\cdot \left(1-p\cdot \left(1-e^{-t/\tau }\right)\right)\right)\cdot e^{-DCR\cdot t} \end{array}$$

(29) $$\begin{array}{cc} F_{min-pois-dcr}\left(t\right) & =1-e^{-\mu_{trig}\cdot \left(1-e^{-t/\tau }\right)-DCR\cdot t} \end{array}$$

(30) $$\begin{array}{cc} f_{min-pois-dcr}\left(t\right) & =\left(\mu_{trig}\cdot \frac{e^{-t/\tau }}{\tau }+DCR\right)\cdot e^{-\mu_{trig}\cdot \left(1-e^{-t/\tau }\right)-DCR\cdot t} \end{array}$$

In the presence of dark counts, the mixed-process arrival-time distributions become proper CDFs and PDFs rather than defective ones because every trial eventually produces either a correlated event or a primary dark event. This follows directly from Equation (4): since $F_{D}\left(t\to \infty \right)=1$, then $F_{min}\left(t\to \infty \right)=1$.

To make the mixed arrival-time distributions more visible in the following figures, we use a relatively high dark count rate, DCR = 1 Mcps, corresponding to a mean time between dark counts of 1000 ns. This value is chosen to be comparable to the exponential decay constant τ = 100 ns.

**Figure 3:**
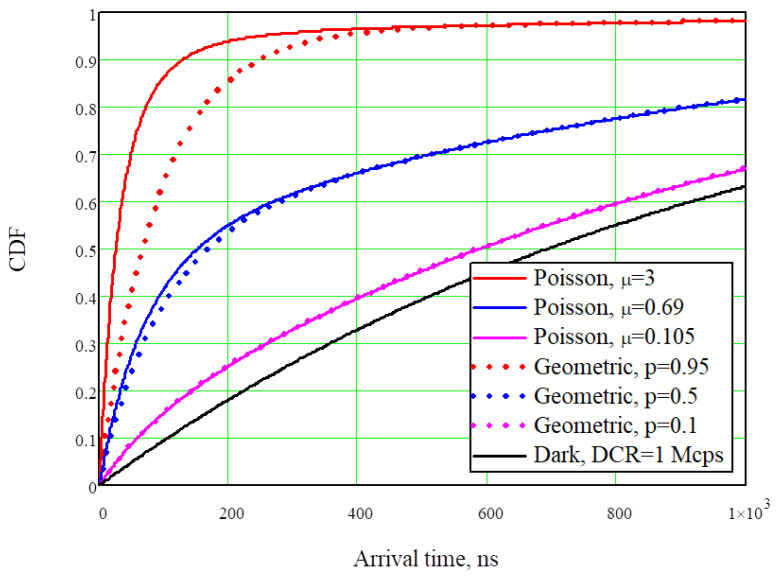
Cumulative distribution functions (CDFs) of the arrival times of the first correlated event from the Poisson branching process, Equation (29), and the single correlated event from the geometric chain process, Equation (27), in the presence of dark counts with DCR = 1 Mcps. The Poisson and geometric processes have the same single-exponential CDF $F_{exp}\left(t\right)$ with a time constant τ = 100 ns, and they have equal correlated event probabilities according to Equations (23) and (24).

As shown in Figure 3, at low probability of correlated events *p* = 0.1, a substantial part of the nearly identical Poisson and geometric CDFs is contributed by dark counts, shown by the “Dark” curve. This occurs because in approximately 90% trials, no correlated event is produced and the majority of events are dark counts. The higher *p*, the lower the relative contribution from dark counts, and at *p* = 0.95, the CDFs in Figure 3 become similar to the corresponding distributions without dark counts shown in Figure 1.

**Figure 4:**
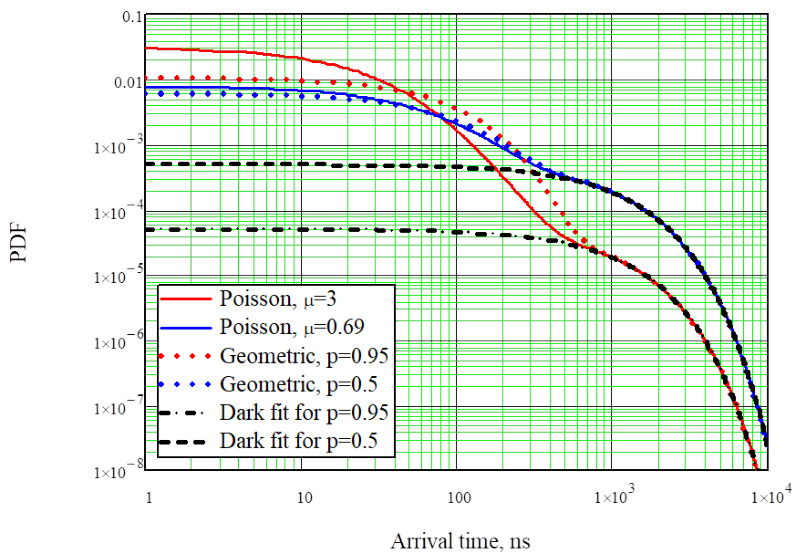
Probability density functions (PDFs) of the arrival times of the first correlated event from the Poisson branching process, Equation (30), and the single correlated event from the geometric chain process, Equation (28), in the presence of dark counts with DCR = 1 Mcps. The Poisson and geometric processes have the same single-exponential PDF $f_{exp}\left(t\right)$ with a time constant τ = 100 ns, and they have equal correlated event probabilities according to Equations (23) and (24).

To emphasize the main features of the PDF plots with high *p* in Figure 4, the case *p* = 0.1 is omitted and the time axis is shown on a logarithmic scale. Dark-count contributions are represented by the “Dark fit” curves. The “Dark fit” function is (1−p)·DCR·exp(−DCR·t), which corresponds to what is typically obtained by fitting the long-time dark-count tail in experimental histograms.

### 3.2. CCDF Approach

A common approach to studying the timing behavior of delayed correlated events is based on the following steps: (A) proper selection of pure single-primary events as measurement triggers, excluding primary events accompanied by prompt crosstalk and secondary events originating from pre-primary avalanches; (B) measurement of a set of $N_{ev}$ arrival times $T_{i}\;\left(i=1,2\ldots N_{ev}\right)$ of the first event after the trigger; (C) construction of a histogram from the set $T_{i}$, representing PDF of the arrival time; and (D) analysis of the histogram using fit functions similar to Equations (28) and (30) either over the full histogram or separately for the long-time dark-count tail and the correlated-event component [22,23,28,31].

However, in our practice, an alternative approach to the analysis of experimental timing data based on empirical CDFs or CCDFs is more straightforward and precise than histogram-based analysis [44]. The construction of histograms requires binning the data into *K* bins, which introduces uncertainty associated with the choice of an optimal bin size. Moreover, binning reduces the original $N_{ev}$ data points to *K* bin points with losses of the original values of $T_{i}$. In contrast, empirical CDF or CCDF is obtained by sorting the set $T_{i}$ in ascending or descending order, assigning them to the X-axis, and assigning normalized indexes $\left(1/N_{ev},2/N_{ev}\ldots 1\right)$ to the Y-axis. Thus, the empirical CDF or CCDF preserves the original timing information. The next steps of the analysis of empirical CCDFs are straightforward. First, the $CCDF_{D}$ (Equation (5)) of the first dark events can be used as a fit function to extract DCR and *p* from the long-time tail of the empirical CCDF. Second, the theoretical $CCDF_{min}$ (Equation (2)) allows us to extract the $CCDF_{C}$ of the correlated events without assuming a specific model of their distribution (except for independence from the dark counts). Note that this model-free extraction is not available in the histogram/PDF approach, because in the theoretical PDF $f_{min}\left(t\right)$ (Equation (7)) the quantities $f_{C}\left(t\right)$, $F_{C}\left(t\right)$, and DCR are mixed in the first (pre-exponential) factor.

**Figure 5:**
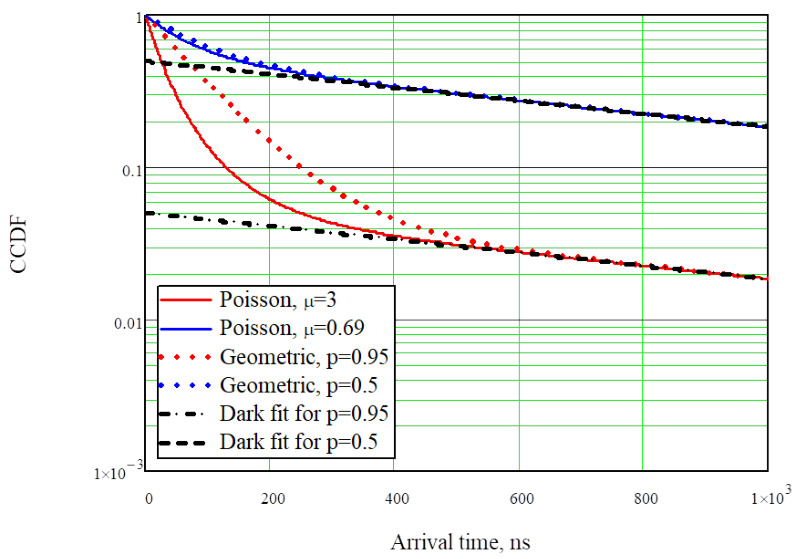
Complementary cumulative distribution function (CCDF = 1 − CDF) corresponding to the PDF plots shown in Figure 4.

The CCDF approach is demonstrated in Figure 5 using the same distributions as in Figure 4. The straight lines labeled “Dark fit” represent $\left(1-p\right)\cdot CCDF_{D}$, and the lines intersect with the CCDF axis at level (1−p). The region above each “Dark fit” line corresponds to the contribution of correlated events, with total probability *p*. An example of experimental data processing for a KETEK SiPM using the CCDF approach is presented in [44].

### 3.3. Studies Related to the Poisson Model

Among studies of arrival-time distributions of delayed correlated events in SiPMs, one of the first papers related to the Poisson distribution of AP events in SiPMs was published in 2008 [22]. The authors presented an expression for the probability of AP in a time interval (t,t+dt), describing it as *“The sum over the Poisson probability accounts for the possibility of having more than one delayed carrier created per avalanche”* (see page 399). This sum is closely related to the differential form of Equation (8) used to express $f_{min}$ (Equation (10)). However, their expression appears to contain an error or misprint in the factor $f_{exp}\left(t\right)$ of the summation, and the sum was not evaluated in closed form.

Another model of the AP time distribution [42] (Equations (3)–(5) of their paper) nearly coincides with Equations (21) and (22). The authors applied Poisson statistics to dark counts and, without discussion, to AP events. However, the definitions of the AP probability used in that work are not consistent. The *“total afterpulse probability”*, denoted by $\epsilon_{AP}$, corresponds to the geometric model of AP events (Equations (19) and (20)), while the *“probability of one or more events until time (t)”*, denoted by $P_{>0}\left(t\right)$, corresponds to the Poisson model of dark and AP events (Equation (15)). This duality leads to a contradiction that can be resolved if we interpret $\epsilon_{AP}$ as $\mu_{trig}$.

A comprehensive study of correlated events using a correct Poisson model for arrival-time distributions was presented at NDIP-2014 and published in 2015 [31]. In that study, Poisson distributions were assumed for the total number of events produced by each type of delayed correlated process and dark counting process. A correct expression for the arrival-time PDF of the first event, similar to Equation (16), was introduced, although without discussion of its order statistic origin.

Therefore, the main expressions derived in Section 2, Equations (13)–(16), are not entirely new. However, they are presented here systematically on probabilistic grounds, with an emphasis on the CDF and CCDF as primary representations that allow separable and experimentally convenient analysis of dark and correlated components.

In 2017, a method was proposed and evaluated that *“allows for a model-independent measurement of the afterpulsing timing distribution and dark noise rate”* in PMTs and SiPMs [43]. The method eliminates the shadowing effect and considers the first event after a properly selected primary event, assuming that both dark counts and delayed correlated events obey Poisson statistics. In this sense, the method is model-independent with respect to the AP timing profile, but it remains dependent on the Poisson-process assumption. Although the method is implemented using histograms, the essential data-processing step is closely related to a CDF-based approach. Specifically, the main expression (Equation (3), page 4) defines the probability $P_{i}$ of obtaining an event in the time bin *i* as the difference between two CDF values. In our notation, replacing the bin times $t_{i}$ and $t_{i+1}$ by *t* and t+dt, their expression becomes a consequence of Equation (15):

(31) $$P_{i}=F_{min}\left(t_{i+1}\right)-F_{min}\left(t_{i}\right)=e^{-N_{sum}\left(t\right)}-e^{-N_{sum}\left(t+dt\right)}=e^{-N_{sum}\left(t\right)}\cdot \left(1-e^{-I_{sum}\left(t\right)\cdot dt}\right)$$

Thus, the method correctly uses a CDF-based formulation, but it does so through histogram binning, which introduces some losses of timing information.

The studies discussed above provided examples of Poisson model validation with respect to experimental histograms of the first event arrival time.

The first-arrival time histograms of the KETEK PM1125 SiPM and Hamamatsu MPPC S10362-11-050C samples were analyzed to determine the characteristics of AP. The Poisson model fit functions appeared to coincide with the histogram data within about 10% tolerance (see Figures 4 and 5, [42]).

The detailed measurements of Hamamatsu MPPC S10362, S12571, and S13360 samples were performed with post-processing separation of delayed CT and AP. The Poisson model was found to be in good agreement for both types of correlated events in a wide range of Poisson means (e.g., from 0.01 to 0.74 for AP), and their PDF timing profiles were found to be defined by diffusion with the relevant time constants (see Figures 8 and 9, and Table 2, [31]).

Other examples of Poisson model verifications were presented in the studies of KETEK PM3350T SiPM (see Figure 4, [44]) and Hamamatsu MPPC for the T2K experiment, similar to the MPPC S10362 series (see Figure 2, [43]).

However, these studies were not focused on a comparative analysis of the Poisson model versus the geometric chain model, especially at high probabilities of correlated events, where we should expect considerable differences, as discussed in Section 3.1. Therefore, a comprehensive verification of the Poisson model is not yet complete and remains to be performed.

## 4. Conclusions

The first-order statistic approach predicts that the arrival-time distribution depends on the number of correlated seeds in a Poisson branch and, consequently, on the probability of correlated events. This contrasts with single-event AP models, such as the geometric chain process, in which the probability and timing profiles are independent. This dependence complicates both the theoretical interpretation and the experimental analysis of correlated processes in SiPMs and SPADs, especially at high probabilities, and may lead to a misinterpretation of the temporal characteristics of afterpulsing and delayed crosstalk.

This analytical model can be a useful tool for analyzing non-exponential and other unusual temporal behavior of silicon and non-silicon Geiger-mode avalanche devices, for example, to explain the formation of “afterpulsing trains”. It may also be especially relevant for studies of radiation-damaged SiPMs with significant lattice defects.

## Data Availability

No new data were created or analyzed in this study. Data sharing is not applicable to this article.
